# Genetic Variability of Koi Herpesvirus *In vitro*—A Natural Event?

**DOI:** 10.3389/fmicb.2017.00982

**Published:** 2017-06-08

**Authors:** Sandro Klafack, Qing Wang, Weiwei Zeng, Yingying Wang, Yingying Li, Shucheng Zheng, Jolanta Kempter, Pei-Yu Lee, Marek Matras, Sven M. Bergmann

**Affiliations:** ^1^Friedrich-Loeffler-Institut, Federal Research Institute for Animal HealthGreifswald-Insel Riems, Germany; ^2^Pearl River Fisheries Research Institute, Chinese Academy of Fishery SciencesGuangzhou, China; ^3^Department of Aquaculture, West Pomeranian University of TechnologySzczecin, Poland; ^4^Department of Research and Development, GeneReach Biotechnology CorporationTaichung, China; ^5^National Veterinary Research InstitutePulawy, Poland

**Keywords:** KHV, VNTR, *Cyprinus carpio*, molecular tracing, CyHV3

## Abstract

Worldwide koi herpesvirus (KHV) causes high mortalities in *Cyprinus carpio* L. aquaculture. So far, it is unknown how the different variants of KHV have developed and how they spread in the fish, but also in the environmental water bodies. Therefore, a phylogenetic method based on variable number of tandem repeats (VNTR) was improved to gain deeper insights into the phylogeny of KHV and its possible worldwide distribution. Moreover, a VNTR-3 qPCR was designed which allows fast virus typing. This study presents a useful method for molecular tracing of diverse KHV types, variants, and lineages.

## Introduction

With a production of four million tons worldwide (http://www.fao.org) common carp (*Cyprinus carpio* L.) is one of the most important fish for aquaculture. Moreover, its ornamental variety, the koi, is one of the most expensive ornamental fish in the world. A severe infection that leads to high mortalities of *Cyprinus carpio* stocks has been reported repeatedly since 1997. This disease is associated with skin lesions and gill necrosis (Bretzinger et al., 1999). The causative agent was first described as an aquatic herpesvirus in Hedrick et al. (2000) and was named koi herpes virus (KHV) after its host. Later, it was given the scientific name Cyprinid herpesvirus 3 (CyHV-3) and was classified as a member of the family *Alloherpesviridae* (Waltzek et al., 2005). With a genome size of 295 kbp, KHV is the largest known herpesvirus. The 11 complete genomes published so far show a very high sequence identity (>99.9%; Aoki et al., 2007; Li et al., 2015; Hammoumi et al., 2016).

To gain more knowledge on the diversity of this virus, a duplex PCR was established (Bigarre et al., 2009) to discriminate between the different virus introductions into Europe. This PCR uses two marker regions to determine the relationship between different KHV variants. Unfortunately, the temporal and spatial resolution of this assay is too low to permit phylogeographical analyses or molecular tracing for origin detection. Subsequently, eight DNA regions with variable number of tandem repeats (VNTRs) were selected and tested as a tool for KHV discrimination (Avarre et al., 2011). Using the fluctuating copy number combined with hierarchical clustering, it was possible to obtain information on the phylogeographical background of some investigated KHV specimens (Avarre et al., 2012).

The present study aimed at designing a new qPCR assay able to distinguish between different variants of the Asian and European lineages. A second goal was to improve the previously described MLVA using different primer pairs covering the eight complete VNTR sequences. Finally, these two methods were applied to trace the variability of four KHV isolates passaged *in vitro* multiple times. With this additional information, a more confident clustering of KHV isolates as well as molecular tracing now seem to be possible.

## Materials and methods

### Cells and replication of KHV isolates

Common carp brain cells (CCB, Neukirch et al., 1999) were grown at 26°C in minimal essential medium with Earls' salts (Invitrogen) supplemented with 10% FBS, 10 mM HEPES, 2.2 g/l NaHCO_3_ (Roth), 1% non-essential amino acids (Biochrom), and 0.12 g/l pyruvic acids sodium salt (Merck). Different KHV isolates (Table 1) were replicated after absorption of virus containing cell culture suspension for 1 h at 26°C onto 24 h-old CCB cells followed by the addition of the necessary volume of cell culture medium.

**Table 1:** Samples used for characterization and discrimination.

**Isolate or specimen**	**Origin**	**Country of origin**
KHV-T	This study	Taiwan
KHV-I	Aoki et al., 2007	Israel
KHV-G1	This study	Germany
KHV-P, F02/05	This study	Poland
KHV-Ch (GZ)	This study	China
F16/12	This study	Germany
F35/13	This study	Germany
F34/13-2	This study	Germany
F34/13-3	This study	Germany
F49/13-2	This study	Germany
KHV-E	This study	England
Ak129	This study	
KHV-V	This study	Vietnam
Italy 197/1	This study	Italy
Italy 197/2	This study	Italy
Italy 197/3	This study	Italy
Italy 204/1	This study	Italy
F11/16	This study	Germany
KHV-GZ11	Dong et al., 2013; Li et al., 2015	China
FL_BAC	Costes et al., 2008	Belgium
CyHV-1	Gilad et al., 2002	Poland
CyHV-2 (specimen)	Gilad et al., 2002	Spain
HVA	Rijsewijk et al., 2005	Germany

### DNA extraction

Extractions of total DNA were done using QIAamp DNA Mini Kit (Qiagen) according to the manufacturer's instructions. Samples were obtained either from cell cultures (500 μl each) or from gill swabs of infected fish. They were incubated overnight at 56°C with 180 μl ATL buffer containing 20 μl proteinase K. Afterwards 200 μl Buffer AL were added and incubated at 70°C for 10 min, followed by addition of 200 μl ethanol. This mixture was applied to the spin columns and centrifuged for 1 min at 800 × g. The flow-through was discarded and the column was washed with 500 μl buffer AW1. After centrifugation for 1 min, the column was washed with 500 μl buffer AW2. The column was spun down and placed in a new tube. DNA was eluted in 50 μl DEPC treated water.

### Primer pairs

Beside the published primer pairs (Bigarre et al., 2009; Avarre et al., 2011), new primer pairs were designed to cover the entire VNTR fragments used for sequence analysis (Table 2) and comparison. For reasons of simplification the VNTR were named according their order in KHV genome.

**Table 2:** Primers used in this study.

**Name**	**Sequence 5′ → 3′**	**References**
VNTR1_for	ACACATCATCAAGAACTTCAGCAAG	This study
VNTR1_rev	CCGTCTTCAGCGTCTCAGT	This study
VNTR2_for	GGCTCACTGCGGAGAACC	This study
VNTR2_rev	GACATGCTGGTCTGGTCCAG	This study
VNTR3_for	GAATACTTCAATCGCATTGTGCC	This study
VNTR3_rev	GCTTGCCACGGTTCCATTAGC	This study
VNTR4_for	CTTGCGCAATGCACTCCG	This study
VNTR4_rev	GCTACTACTGCTGCTGCTGAG	This study
VNTR5_for	GTATAACAGCCGCCACGAATC	This study
VNTR5_rev	CAATCAGCAGCGACGCTAAG	This study
VNTR6_for	CAAATGGCGCAGCGCTG	This study
VNTR6_rev	CGATGTCCGACGCCTTTCT	This study
VNTR7_for	CTCTGGTTCTGGTTCTGGCTC	This study
VNTR7_rev	ACATGATGGTCAGGCCAGTC	This study
VNTR8_for	CAGAGCGGTTCTGCCTTTG	This study
VNTR8_rev	CCAACCAGACCCAAGAAGCAG	This study
oPVP53	CTACTCAGGAGCCATCATCG	Bigarre et al., 2009
oPVP54	AGGACTTGGTAGGTGCCTCC	Bigarre et al., 2009
oPVP55	GCTCATTTTAGCGCTTCTGTG	Bigarre et al., 2009
oPVP56	CGCTGCCTACCCAATTCGCT	Bigarre et al., 2009
120160 for	CAACAGTACAACCACAACATCGA	Avarre et al., 2011
120160 rev	GGTAACATTGGCGGTAGAACTA	Avarre et al., 2011

### PCRs for identification and characterization of the KHV isolates and specimens

Initially, all KHV isolates and specimens obtained from carp or koi were examined by qPCR as previously described (Gilad et al., 2004, modified according to Bergmann et al., 2010). Additionally, duplex PCR (Bigarre et al., 2009) was performed for primary characterization and classification into the Asian or European KHV lineage. All PCR products were separated in 1.5% agarose gels.

### VNTR amplification

The eight VNTRs described by Avarre et al. (2011) were amplified using the Phusion Green High-Fidelity DNA Polymerase (Thermo Scientific) according to the manufacturer's instructions. The different VNTRs were separated in agarose gels and the resulting bands were clipped. DNAs from gel blocks were extracted using the QIAquick Gel Extraction Kit (Qiagen), and 12.5 μg of each VNTRs were sequenced with either the forward or reverse primer using the BigDye Terminator v1.1 Cycle Sequencing Kit (Thermo Scientific) following the manufacturer's instructions.

### Sequence data analysis

Sequence information was imported into Geneious (Geneious 9.0.5, Kearse et al., 2012) and all eight VNTRs of each KHV sample were combined into one sequence. These concatenated sequences were aligned using ClustalW (Larkin et al., 2007; Supplementary Data Sheet 1). Phylogenetic trees were constructed with IQtree and ultrafast bootstrap (*B* = 1000; Minh et al., 2013; Nguyen et al., 2015; Chernomor et al., 2016). Nod supporting values are approximately unbiased (au) *p*-values. IQtree chose best fitting model for genomic data, Jukes-Cantor. Same model was used for VNTR data. Hierarchical clustering of duplex PCR data was done with RStudio (R Development Core Team, 2010; Rstudio Team, 2015). As comparison for phylogenetic analysis, the full genome sequences of KHV-I (GenBank: DQ177346.1), AK129 (unpublished), KHV-GZ11 (GenBank: KJ627438.1), KHV-U (GenBank: DQ657948.1), NC_009127 (NCBI: NC_009127.1), FL_BAC (GenBank: KP343683.1), KHV-J (GenBank: AP008984.1), and KHV-T (unpublished) were used.

### Establishment of a new TaqMan qPCR based on VNTR 3 sequence

Based on VNTR 3, a TaqMan qPCR was established using two different probes. One probe covered the sequences shown in the VNTR 3 of the European KHV lineage and the other probe covered sequences of the same VNTR, but only the Asian KHV lineage was selected (Table 3). This was combined with sequence data obtained with forward and reverse primers (Table 2). For TaqMan qPCR, the QuantiTect Multiplex PCR Kit (Qiagen) was used following the manufacturer's instructions. Initially a denaturation step at 95°C for 15 min was started followed by 42 cycles with thermal profile: 95°C for 1 min, 60°C for 30 s and 72°C for 30 s. All samples were tested in the presence of three negative controls (water). The threshold was set to C_*q*_ = 39 (~1–5 particles/ml, Bergmann et al., 2010) for discrimination between positive and false positive samples.

**Table 3:** Probes for the TaqMan PCR.

**Probe**	**Sequence**	**Lineage type**
a (Asia)	….CAATACCACCAGCGCATCCAAC.…	Asia
e (European)	….CCGGTCAGGTGCGCGCTCACTCAGCGCATCCA…	Europe

## Results

### VNTR-3 qPCR for KHV discrimination

**Figure 1:**
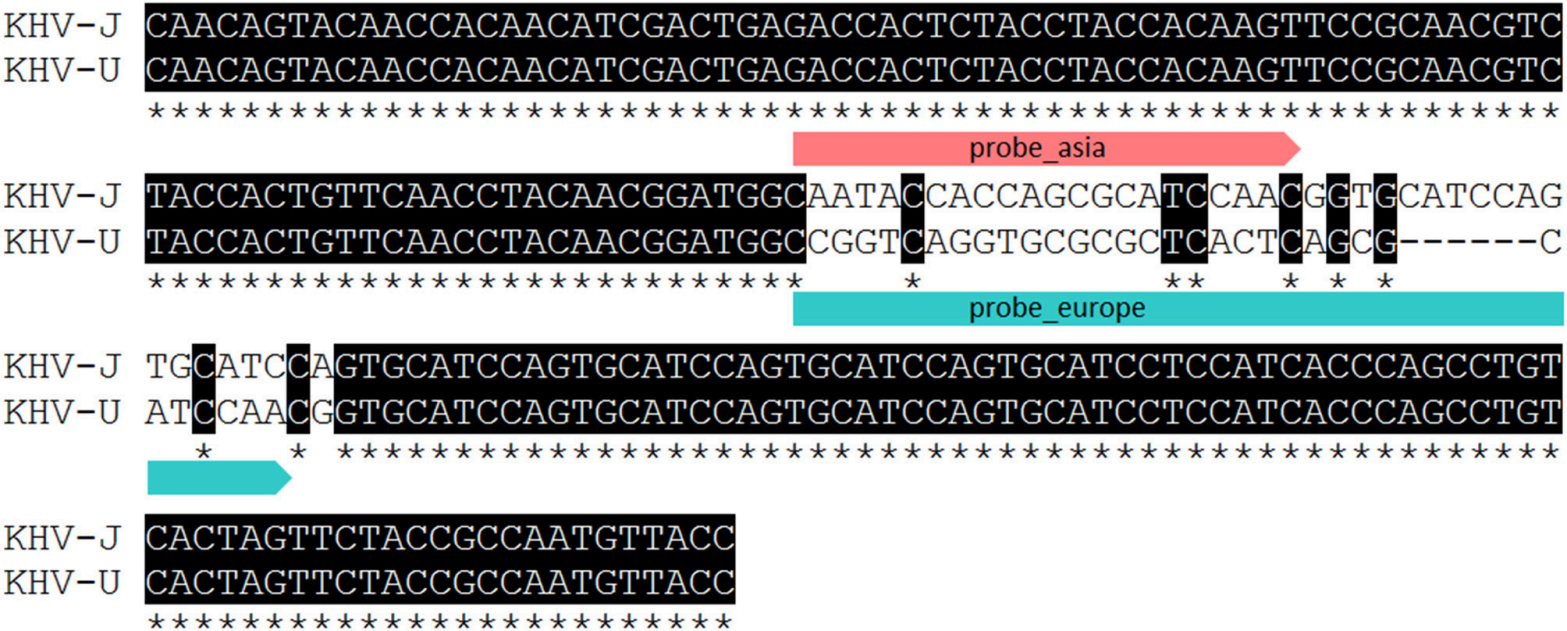
Partial alignment of VNTR 3 presenting binding sites of qPCR probes used for lineage determination and discrimination. Asterisk indicating identical nucleotides.

Identification and characterization of the isolates and specimens (Table 4) were carried out with the newly designed qPCR based on VNTR 3 sequences (Table 3) and were compared to the results obtained with the diagnostic qPCR (Gilad et al., 2004) as well as to the results from the duplex PCR (Bigarre et al., 2009; Table 5). The third VNTR possesses an eye-catching genetic feature to roughly discriminate between Asian and European variants of KHV. Therefore, a qPCR was designed based on this sequence (Figure 1 and Table 3). A control DNA from CCB cells was used to exclude a cross-reaction of the PCR with carp DNA. Specificity was also tested with other herpesviruses, in particular CyHV-1, CyHV-2, and herpesvirus anguillae (HVA), as well as with the virus causing koi sleepy disease, the carp edema virus (CEV). None of the tested controls became positive by qPCRs, in spite of a faint signal for CyHV-2 with a mean *C*_q_-value of 40.1 which was considered negative, as the detection limit was set at a *C*_q_-value of 39. All tested KHV samples were considered to be positive. Unexpected results were obtained for a Chinese isolate (KHV-Ch, Dr. Wang Qing), for a sample from Poland and one diagnostic sample from koi in Germany (F11/16). These three samples showed positive signals for both, the Asian and the European lineage. Most interestingly, diagnostic sample F34/13-2, identified as a so-called atypically reacting KHV, did not react positive with the diagnostic qPCR (Gilad et al., 2004) but was positive with the newly designed VNTR-3 qPCR. Finally, according to the VNTR-3 qPCR results KHV-V that was identified in Vietnam belongs to the European lineage.

**Table 4:** Real-time PCR results from different KHV samples and controls.

**Sample**	**Probe Asia (a)**		**Probe Europe (e)**		**Probe KHV (Gilad et al.,** **2002****)**	
	**mean *C_q_***	sd^*^C_q_	**mean *C_q_***	**sd *C_q_***	**mean *C_q_***	**sd *C_q_***
CyHV-1	−	−	−	−	−	–
CyHV-2	40.11	1.19	−	−	−	–
HVA	−	−	−	−	−	–
CEV	−	−	−	−	−	–
carp	−	−	−	−	−	–
KHV-E	−	−	26.00	0.20	25.32	0.17
KHV-G1	−	−	10.96	0.08	10.61	0.46
KHV-Ch	31.71	4.87	34.56	−	39.59	–
KHV-V	−	−	22.43	0.02	21.98	0.03
Ak129	−	−	10.08	0.11	10.16	0.43
KHV-T	17.72	0.14	−	−	13.48	0.25
F16/12	−	−	−	−	−	–
F35/13	−	−	−	−	39.82	-
F34/13-2	40.62	–	37.02	−	−	–
F34/13-3	−	−	−	−	−	–
F49/12-2	−	−	−	−	−	–
Italy 197/1	−	−	25.79	0.34	25.51	0.02
Italy 197/2	−	−	25.07	0.18	24.79	0.16
Italy 197/3	−	−	24.63	0.06	24.41	0.21
Italy 204/1	−	−	25.75	0.08	25.15	0.03
F02/05	32.93	0.04	27.83	0.14	20.95	0.08
F11/16	32.39	0.27	37.81	−	−	–

* *sd, standard deviation*.

*Gray values were above detection threshold (C_q_ = 39). Missing values were indicated by –, absent values were caused by no amplification in PCR or by less values for calculating standard deviation*.

**Table 5:** Results for different KHV properties.

**Name**	**Origin**	**Duplex PCR**		**Lineage**
		**Marker I**	**Marker II**	**qPCR**
KHV-T	Asia	+ +	+	Asia
KHV-T Passage 1	Laboratory	+ +	+	Asia
KHV-T Passage 25	Laboratory	+ +	+	Asia
KHV-T Passage 51	Laboratory	+ +	+	Asia
KHV-T Passage 78	Laboratory	+ +	+	Asia
KHV-T Passage 99	Laboratory	+ +	+	Asia
KHV-E	Europe	−−	−	Europe
KHV-E Passage 4	Laboratory	−−	−	Europe
KHV-E Passage 25	Laboratory	−−	−	Europe
KHV-E Passage 51	Laboratory	−−	−	Europe
KHV-E Passage 51	Laboratory	−−	−	Europe
KHV-E Passage 78	Laboratory	+ +	+	Asia/Europe
KHV-E Passage 99	Laboratory	+ +	+	Asia
KHV-Israel Passage 4	Laboratory	−−	−	Europe
KHV-Israel Passage 25	Laboratory	−−	−	Europe
KHV-Israel Passage 51	Laboratory	−−	−	Europe
KHV-Israel Passage 78	Laboratory	+ +	+	Asia/Europe
KHV-Israel Passage 99	Laboratory	−−	−	Europe
KHV-G1	Europe	−−	−	Europe
KHV-G1 Passage 4	Laboratory	−−	−	Europe
KHV-G1 Passage 25	Laboratory	+ +	+	Asia
KHV-G1 Passage 51	Laboratory	+ +	+	Asia
KHV-G1 Passage 78	Laboratory	+ +	+	Asia
KHV-G1 Passage 99	Laboratory	+ +	+	Asia

### Comparison of results from duplex PCR vs. the improved VNTR analysis

Specificity of the eight new VNTR primer pairs was tested with DNA obtained from other members of the genus *Cyprinivirus*: CyHV-1, CyHV-2, and *herpesvirus anguillae* (HVA). Only with the primers of VNTR 2 a signal was detected from CyHV-1 and CyHV-2 DNA (Supplementary Image 1). No signal at all was detected using DNA from HVA.

By sequence analysis of VNTRs, different types of KHV (Asian and European lineages) were found in the same isolate or specimen. Moreover, the results obtained from VNTR sequences are comparable and confirmed the results from genome sequences or the results obtained with the duplex PCR (Supplementary Table 1).

**Figure 2:**
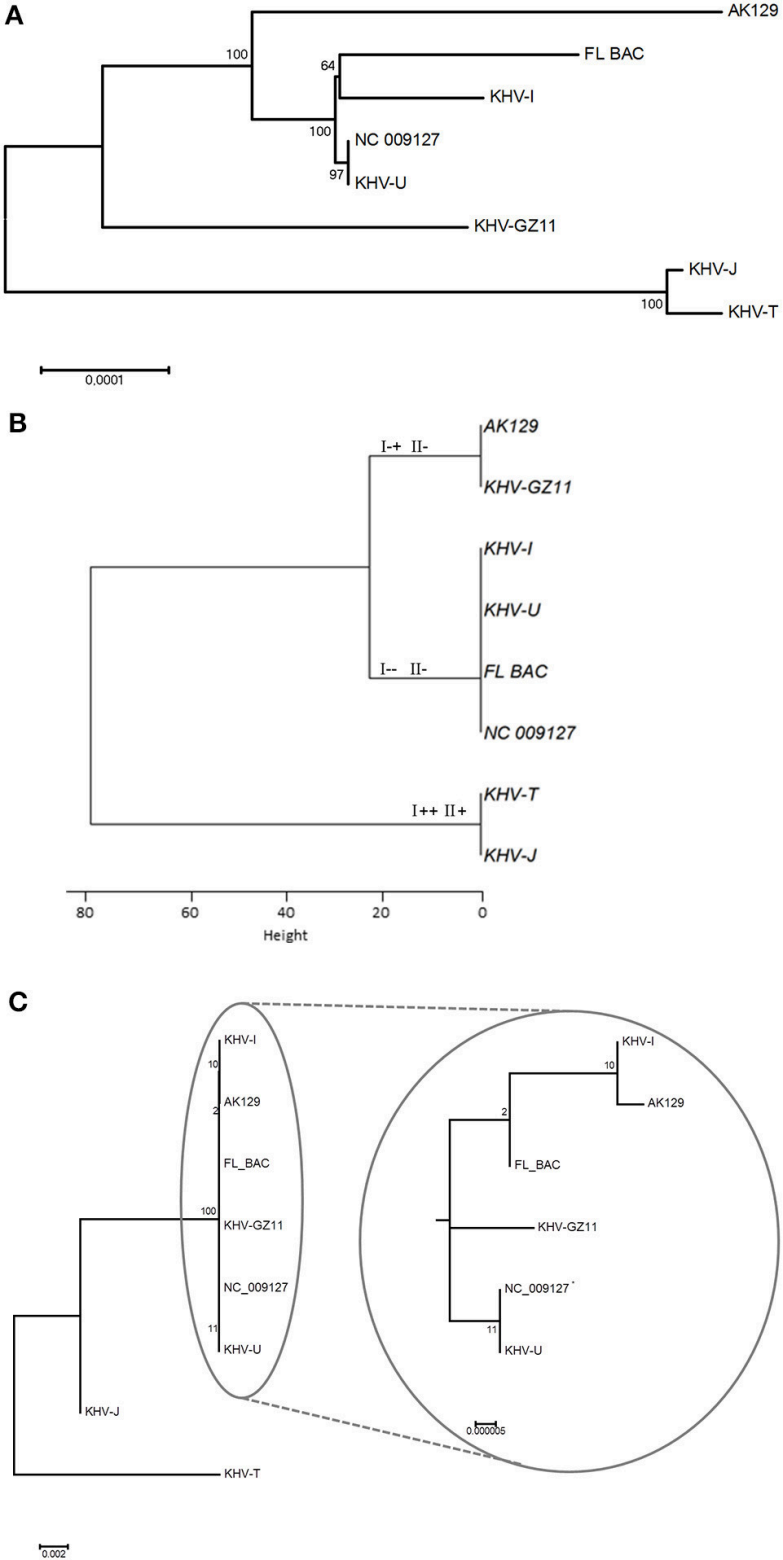
Phylogenetic trees based on different methods. **(A)** Presents phylogeny based on whole genome sequences, while **(B)** shows information gained by duplex PCR. Genotypes according to Bigarre et al. (2009) were added at each branch. VNTR sequencing was used to generate the phylogenetic tree in **(C)**. Because of 100% identity of NC_009127 and KHV-U, NC_009127 was included as internal control.

KHV isolates or specimens from Japan and Taiwan formed one cluster, known as the Asian lineage of KHV. This cluster can be distinguished using all three methods (Figures 2A–C). In contrast, the other six viruses cluster differently with each method.

The topology of the phylogenetic tree based on genome sequences (Figure 2A) and the tree based on VNTR sequencing (Figure 2C) were comparable. Additionally, the genomic data from GZ11 suggest an intermediate mode of the virus between the two known lineages.

Due to increasing sample sizes and much more complete genomic data, the phylogenetic diversity can be investigated in greater detail. Analysis with sequences obtained from MLVA and/or VNTR sequencing additionally shows different (sub)groups within the two major lineages which cannot be identified by duplex PCR.

### Monitoring of KHV isolates from Taiwan to England after *in vitro* passages

**Figure 3:**
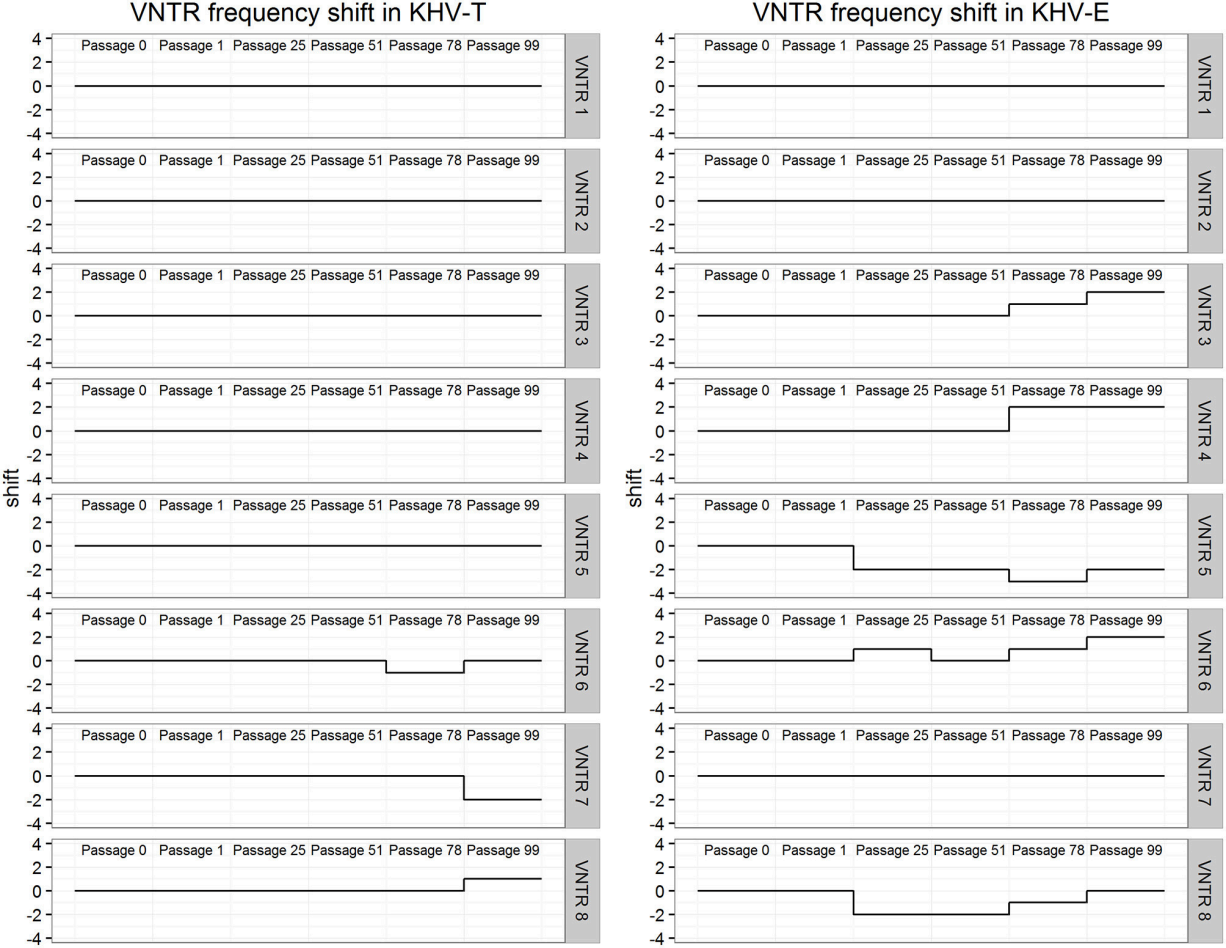
Differences in VNTR frequency over long-term virus passages of KHV-T and KHV-E.

In order to investigate possible mutations of the virus over time in cell cultures, two samples of different origin (KHV-T and KHV-E) were monitored over 100 passages onto CCB cells at 20°C. For this purpose, samples were analyzed periodically with the three. The results revealed a constant evolutionary pressure even within a consistent environment. Plotting the VNTR frequencies over time (Figure 3) reveals the evolutionary pressure. KHV-T only showed changes in sequences of VNTRs 6, 7, and 8. While in the KHV-T VNTRs 6 and 7 one or two repeats were not detectable, in VNTR 8 a repeat was added. Comparing these findings with the VNTR frequencies of KHV-E it was shown that changes were visible in VNTRs 3–6 and in VNTR 8. Generally, the European KHV from England (KHV-E) showed more variations than the Asian type KHV-T.

## Discussion

**Figure 4:**
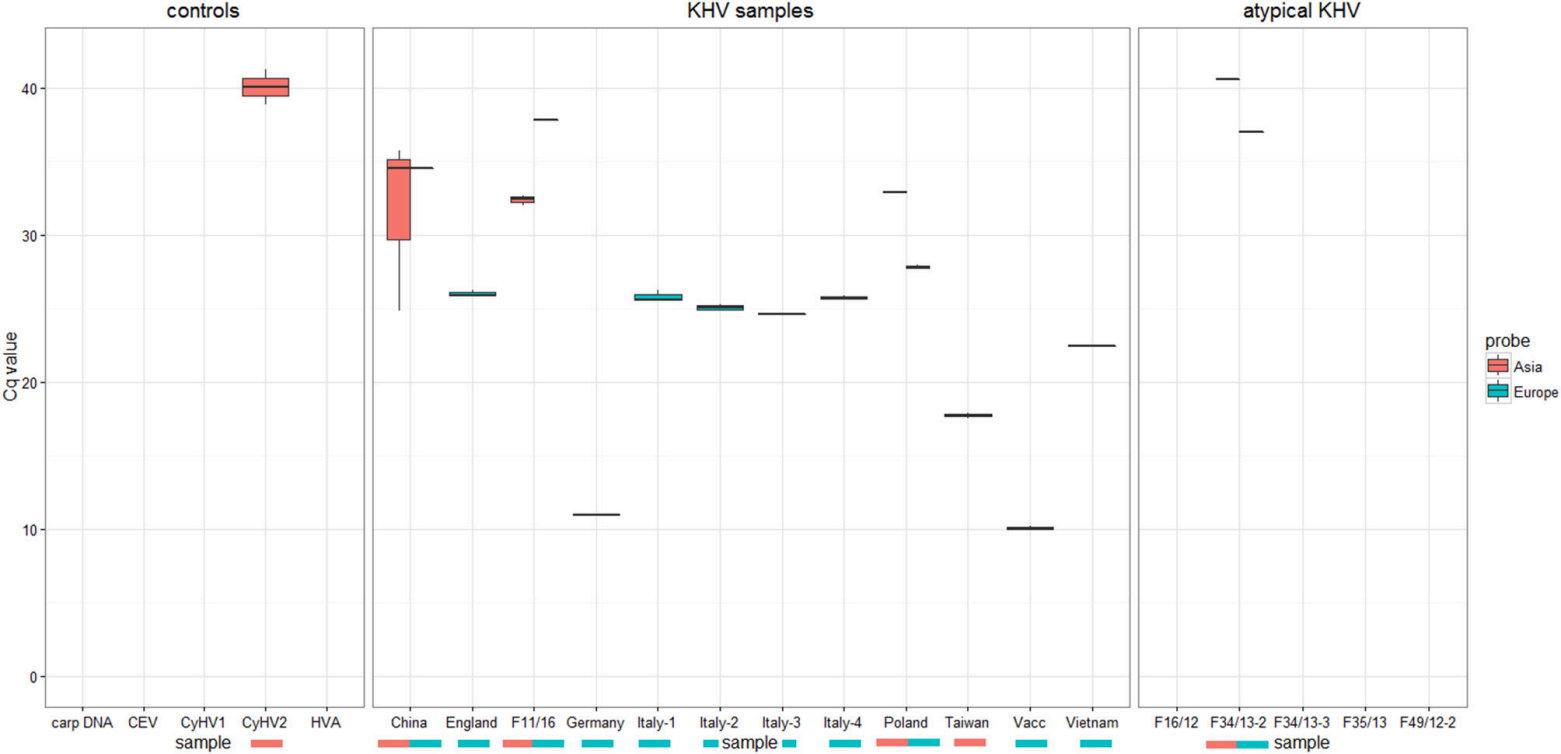
Results of KHV typing by real-time PCR using specific probes either Asian types or European KHV variants. No detection of CyHV1, CEV, HVA, or carp DNA was recognized.

For KHV, 11 complete sequenced genomes are now available in the databases. While full length genomes are compulsory for phylogeographical investigations, the costs for next-generation sequencing, and the lack of handiness for the analysis of raw sequence data still hamper wide genome-based phylogeographical studies. To reduce these drawbacks, the duplex PCR was designed to distinguish between different types, variations or possible introductions of KHV to Europe (Bigarre et al., 2009). Unfortunately, this method was able to detect four different variations or types of KHV only. To overcome this possible gap, an MLVA-based method was established and included in this study. Based on eight different VNTR sequences, it was possible to distinguish more precisely the phylogeny of KHV. It was decided to use and sequence the VNTRs further on, even though they represent only 0.56% of the entire KHV genome. Additionally, by increasing the numbers of tested virus isolates and/or specimens by duplex PCR and VNTR-3 qPCR analyses, the informative value of the VNTRs will become strikingly more precise. In the case of the complete genome of the Chinese KHV isolate GZ11, an intermediate status appears in comparison to the European and Asian lineages. Complete genome analysis indicated that this Chinese virus had a phylogenetic position between European viruses like KHV-U or KHV-I and Asian types like KHV-J or KHV-T Considering the information obtained by duplex PCR, the isolate GZ 11 shares the same genotype as the attenuated AK-129. However, the phylogenies obtained from whole genome sequences and VNTR data indicate that GZ11 does not cluster together with AK-129. This shows that the markers used in the duplex PCR are not informative enough and may lead to incorrect conclusions. Detecting different variants of KHV in a single isolate or specimen is a major challenge for diagnostics. Combating the spread of KHV depends on these efforts. Moreover, different types of KHV may possess different pathogenic features which determine the viability of a population. Tracking Asian and European lineages by qPCR, the first cut was done for applied diagnostics. The importance is shown by the data (Figure 4) obtained with samples from KHVD outbreaks and with experimental samples. The most interesting samples are those which include both, the European and the Asian lineage, e.g., F02/05 from Poland (KHV-P), “atypically reacting” samples F34/13-2 and F11/16 from Germany and the KHV isolate from China (KHV-Ch, named also GZ for Guangzhou). These samples seem to have both features. This can either be the result of mixed infections with different KHV lineages in one isolate or of *in vivo* replication of the infecting agent that generates different variations or types, thereby increasing the likeliness of infection of a fish or a population at the same time. Mixed infections with differential KHV have already been reported, although further investigations are required to elucidate their ecological significance (Sunarto et al., 2011; Avarre et al., 2012; Li et al., 2015). However, a more important point is that this VNTR-3 qPCR neither detected CyHV-1, CyHV-2 nor HVA or CEV. Especially, discrimination of CEV from KHV is important because of the very similar symptoms in carp they cause and their cumulated emergence (Miyazaki et al., 2005; Jung-Schroers et al., 2015). The fact that CyHV-1 and CyHV-2 could only be detected with VNTR-2 primers indicates that the improved MLVA method does not recognize DNAs of other Cypriniviruses. This is especially important because of the high similarity of the three cyprinid viruses to each other as well as to the other species member HVA. By reducing the low specificity of the primer pair for VNTR-2 PCR, it should be possible to perform phylogenetic studies even with samples comprising more than one *Cyprinivirus* like KHV, carp pox virus, goldfish herpesvirus and/or HVA as a PAN-*Cyprinivirus* PCR.

The improved VNTR analysis was suitable to monitor the effects of KHV passages. During the 100 passages of KHV-T and KHV-E as well as KHV-Israel and KHV-G1 onto CCB cells at 20°C, it was possible to observe different changes in the VNTR sequences. Moreover, it was possible to detect a shift in lineage affiliation of KHV-E. After passage 78, the virus showed features of Asian KHV variants by both VNTR-3 qPCR and duplex PCR.

It is known from bacteria that VNTRs play an important role in adaptation, especially in relation to pathogenesis and virulence (van Belkum et al., 1997). Here, since the VNTRs are located in putative open reading frames, varying number of repeats may be a way to adapt to changing conditions by modifying the structural properties (i.e., transmembrane domains) of the corresponding proteins. Furthermore, it was found that changes in lineage affiliation of European KHV may occur. By both duplex PCR and VNTR-3 qPCR, shifts were visible. All three European-typed viruses shifted into the Asian lineage at different time points. Surprisingly, KHV-I was able to shift back, which was confirmed by duplex PCR. While with duplex PCR it was only possible to detect the lineage shift, only the VNTR-3 qPCR made it possible to see that in some cases, both Asian and European types were present. This indicated that these genetic variations were most likely due to the presence of several virus variations in the sample with one overcoming the other(s), of course the most virulent and fastest growing isolate. It is striking that only the European samples shifted into the other lineage but not the used Asian isolate KHV-T. In this case it must be clarified using other Asian type isolates that they are more stable in passaging. Nevertheless, most genetic variations were observed in European viruses. This may indicate that the Asian type is better adapted than the European types which may underline the fact that KHV was introduced to Europe from Asia. Since its introduction to Europe it needs to struggle with new ecological conditions like water temperature, seasonal cycles and changes, and different fish populations, which leads to an ongoing adaptation process. Moreover the phylogenic position of KHV GZ11 supports this possibility (Figure 2A). Because of its intermediate status, KHV GZ11 seems to be on the half way between the Asian cluster and the other European samples like KHV-U or KHV-I. But it still belongs to the European lineage.

Now it should be possible to detect different types of KHV more rapidly as well as to find possible subtypes or mixtures of KHV variants in a sample. However, this method is not yet an alternative for KHV diagnostics. It may however serve as an additional tool for deeper insights into KHV, its adaptation, its changes, and its distribution in these variations.

Beyond the phylogenetic analyses it might be possible to predict the virulence of a KHV with this method, if the appropriate marker genes are used. However, studies need to be performed to gain deeper insights into the virulence of KHV, especially which genes or modifications are important for high or low virulent virus variants.

Finally, this method does not only permit to monitor KHV in the wild and in carp or koi pond cultures but also to use it in cases of KHVD in these fish. Moreover, this technique has the potential to trace back introduced KHV inducing KHVD to their source. This might become useful, if Australia realizes its plan to eradicate or control the common carp population by using KHV (http://www.csiro.au).

## Author contributions

SK and SB planned and performed experimental work. SK, QW, WZ, YW, YL, SZ, JK, PL, MM, and SB discussed the results.

### Conflict of interest statement

The authors declare that the research was conducted in the absence of any commercial or financial relationships that could be construed as a potential conflict of interest.
