# Supplementary figures and images for: Genetic Variability of Koi Herpesvirus In vitro—A Natural Event?
Source: Front Microbiol. 2017 Jun 8;8:982. doi: 10.3389/fmicb.2017.00982 (PMC5462989; doi:10.3389/fmicb.2017.00982)

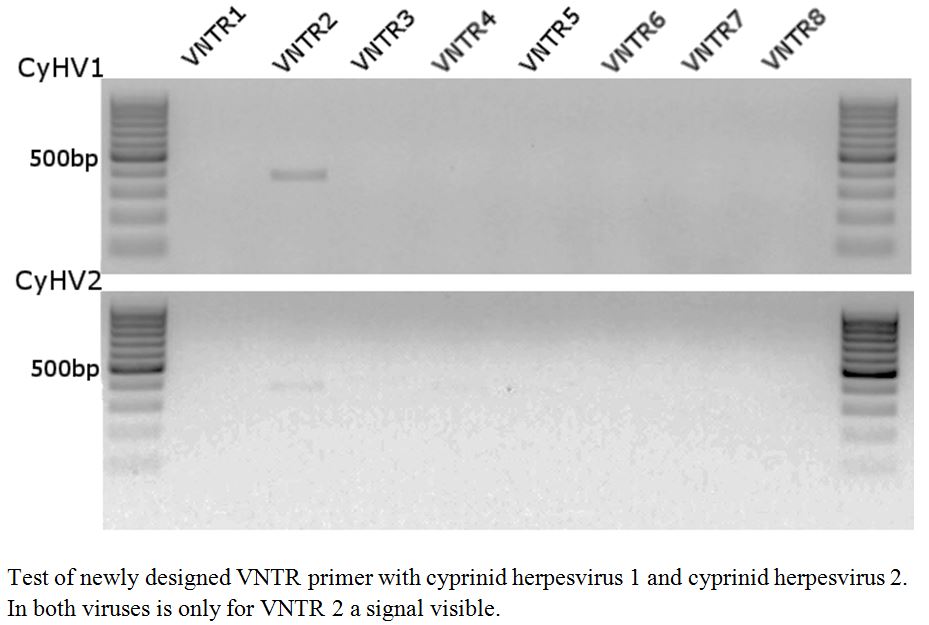

Supplement: Supplementary file 3 [file Image1.JPEG]
